# Digitally Guided Direct Composite Injection Technique with a Bi-layer Clear Mini-Index for the Management of Extensive Occlusal Caries in a Pediatric Patient: A Case Report

**DOI:** 10.3290/j.jad.b4515527

**Published:** 2023-10-16

**Authors:** Keiichi Hosaka, Antonin Tichy, Monica Yamauti, Keiichiro Watanabe, Kohei Kamoi, Kazuhide Yonekura, Richard Foxton, Masatoshi Nakajima

**Affiliations:** a Professor, Department of Regenerative Dentistry, Tokushima University Graduate School of Biomedical Sciences, and Division of Interdisciplinary Research for Medicine and Photonics, Institute of Post LED Photonics, Tokushima University, Tokushima, Japan; Visiting Scientist, Department of Electrical Engineering and Computer Science, Microsystems Technology Laboratories, Massachusetts Institute of Technology, Cambridge, MA, USA. Protocol development, clinical case operator, wrote the manuscript, project administration.; b Assistant Professor, Institute of Dental Medicine, First Faculty of Medicine of the Charles University and General University Hospital, Prague, Czech Republic. Protocol development, wrote the manuscript.; c Associate Professor, Operative Dentistry, Graduate School of Dental Medicine, Hokkaido University, Hokkaido, Japan. Protocol development, wrote the manuscript.; d Assistant Professor, Orthodontics and Dentofacial Orthopedics, Tokushima University Graduate School of Biomedical Sciences, Tokushima, Japan. Protocol development, wrote the manuscript.; e Registered Dental Technician, Department of Dental Laboratory, Tokushima University Hospital, Tokushima, Japan. Protocol development, investigation.; f Assistant Professor, Department of Regenerative Dental Medicine, Tokushima University Graduate School of Biomedical Sciences, and Division of Interdisciplinary Research for Medicine and Photonics, Institute of Post-LED Photonics, Tokushima University, Tokushima, Japan. Protocol development, investigation.; g Lecturer, Faculty of Dentistry, Oral & Craniofacial Sciences, King’s College London, London, UK. Protocol development, wrote the manuscript.; h Visiting Professor, Department of Regenerative Dental Medicine, Tokushima University Graduate School of Biomedical Sciences, Tokushima, Japan. Protocol development, project administration.

**Keywords:** bi-layer clear mini-index, digital workflow, direct composite restoration, endodontically treated tooth, injection technique

## Abstract

**Purpose:**

This case report presents a direct composite inverse injection technique using a bi-layer clear mini-index fabricated with a digital workflow to restore extensive posterior occlusal cavities in a 13-year-old patient.

**Materials and Methods:**

After a root canal treatment in the right mandibular first molar and step-wise excavation of deep caries in the left mandibular first molar, the extensive occlusal restorations were digitally designed using CAD software, upon which digital wax-ups were 3D-printed. Bi-layer clear mini-indices consisting of a hard outer plastic layer and an elastic inner silicone layer were prepared from the 3D-printed cast. The bonding surfaces were deproteinized using a 6% sodium hypochlorite solution, and an antioxidant (Clearfil DC Activator; Kuraray Noritake) was utilized to improve the dentin bonding durability of a 2-step self-etch adhesive (Clearfil SE Bond 2; Kuraray Noritake). Subsequently, a highly filled universal-shade flowable resin composite (RC) was incrementally placed into the cavities. To create the final occlusal morphology, the same RC was inversely injected through the opening of the bi-layer indices.

**Results:**

The workflow was feasible, and the occlusal cavities were efficiently restored using the injection technique. Occlusal carving and adjustments of the morphology were not necessary, leading to less chair time. At the 1-year follow-up, the clinical outcome was excellent.

**Conclusion:**

The injection technique with a bi-layer clear mini-index accurately translated the digital wax-ups into large, final restorations. Precise morphology and shortened chair time enhanced patient satisfaction, but at the expense of multiple visits.

Untreated dental caries in permanent and deciduous teeth is the most prevalent of all medical and dental diseases, whereby 2.5 billion people worldwide are expected to be affected.^4,16^ In deep caries, vital pulp therapy enables preservation of the vitality and function of the dental pulp.^1,11^ However, indirect restorations, such as a partial crowns, full crowns, or endo-crowns, are often required due to the extensive loss of dental hard tissues.^8,44^ On the other hand, the evolution of adhesives and resin composites (RCs) in recent years has expanded their range of applications.^26,38^ Direct RC restorations are frequently provided as an esthetic and functional treatment even in large cavities, including restorations of endodontically treated teeth.

While direct RC restorations follow the minimal intervention dentistry (MID) concept,^12,45^ their placement may be technique sensitive and time consuming. Therefore, several techniques have been introduced to facilitate their placement, such as the stamp technique, which is suitable for posterior occlusal restorations. The stamp technique copies the original tooth morphology using a light-curing resin-based material applied on the occlusal surface prior to preparation.^2,24,31^ The stamp is then pressed against the final composite increment, avoiding the need to sculpt the occlusal surface. The silicone index technique entails a negative anatomical form prepared on the dentition prior to tooth preparation or on a diagnostic wax-up model that simulates the morphology of the final restoration. For extensive anterior restorations, the silicone index technique can assist the reconstruction of the palatal surface, which facilitates the incremental placement of RC and speeds up the restorative procedure.^25^ Recently, the inverse injection technique was introduced, utilizing a highly filled, flowable RC and a highly clear (transparent) silicone index pressed against the dentition.^41,42^ In this technique, the flowable RC is inversely injected through an access hole prepared in the clear silicone index, allowing for an efficient and accurate placement of the flowable RC even in complicated esthetic cases and full-mouth reconstructions.^7,14,33,41,42,47^

Digital technologies have revolutionized restorative dentistry, prosthodontics, orthodontics, oral surgery, and implant dentistry. The integration of intra-oral and laboratory scanners, computer-aided design (CAD), and computer-aided manufacturing (CAM) has transformed dental practices and laboratories, providing many benefits. A digital workflow is increasingly used for direct CR restorations as well,^32,34^ especially for the injection technique in anterior teeth.^6,15,19,21,32,46,48^ However, to the best of our knowledge, digitally guided RC restoration of extensive posterior cavities, including endodontically treated teeth, using the injection technique has yet to be reported. Therefore, this article presents a digitally guided direct composite injection technique for extensive cavities after root canal treatment and step-wise excavation. We developed an improved single-tooth-sized bi-layer clear index, composed of an outer hard plastic and an inner elastic silicone layer, to adapt it to the target tooth, even with single-tooth rubber-dam isolation.

## Case Report

**Fig 1:**
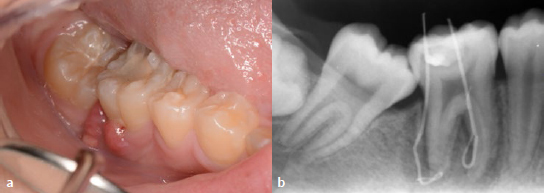
Preoperative examination of the tooth 46. a) The patient complained of gingival inflammation. b) Preoperative radiograph with inserted gutta-percha in sinus tracts.

**Fig 2:**
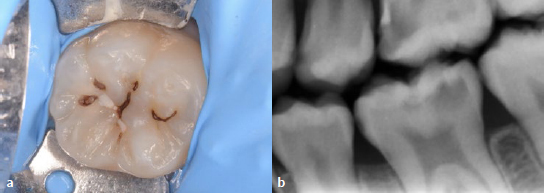
Preoperative examination of the tooth 36. a) Preoperative occlusal view. b) A deep carious lesion was detected in the bitewing.

A 13-year-old female patient was referred to the Department of Operative Dentistry, Tokushima University Hospital, from an orthodontic clinic because of a high caries risk according to the CAMBRA (Caries Management by Risk Assessment) system. Upon examination, the patient presented with a swelling of the gingival tissue surrounding the mandibular right first molar (tooth 46), which had a defective RC restoration with secondary caries (Fig 1a). The patient did not have any pain. Cold sensitivity testing was performed, and no response was elicited. Periodontal pocket depths were 3 mm around the tooth. Intraoral radiographs were taken with gutta-percha points in the sinus tracts (Fig 1b), and chronic apical periodontitis was diagnosed. Furthermore, a deep carious lesion was also diagnosed in the left mandibular first molar (tooth 36) (Fig 2). Sensitivity testing gave a physiological result, and there were no symptoms.

### Root Canal Treatment (tooth 46) and Step-wise Excavation (Tooth 36)

The previous RC restoration and secondary caries in tooth 46 were removed under rubber-dam isolation, without the need for local anesthesia. A caries detector dye solution (Caries Check, Nippon Shika Yakuhin; Shimonoseki, Japan) was used to verify complete caries removal. Subsequently, root canal treatment was carried out. A pre-mixed calcium hydroxide paste (Calcipex II, Nippon Shika Yakuhin) was applied into the root canals, and the access cavity was temporarily sealed with a glass-ionomer cement (Base Cement, Shofu; Kyoto, Japan). After 3 weeks, the sinus tract had healed and radiographic examination revealed a reduction in the periapical radiolucency, so the root canals of tooth 46 were obturated. This was followed by the provisional placement of a glass-ionomer cement (Base Cement, Shofu).

For tooth 36, step-wise excavation was undertaken to manage the deep occlusal caries. While peripheral dentin was excavated non-selectively to ensure a durable seal of the lesion, selective caries removal of soft dentin using a spoon excavator was performed at the pulpal aspect of the cavity. Following a protocol by Momoi et al,^30^ a polycarboxylate cement containing a tannin/fluoride compound (HY-Bond Temporary Cement Soft, Shofu) was subsequently placed in the cavity, with re-entry planned after three months. The mesial incipient caries lesion found in the radiograph was only treated using a fluoride varnish, as it was not cavitated and the radiolucency was limited to the outer half of enamel. Finally, a full-mouth digital impression was taken using an intraoral scanner (Cerec Primescan, Dentsply Sirona; Bensheim, Germany). Given the sufficient amount of remaining dental hard tissues on teeth 36 and 46, it was planned to use RC for the final restoration in both teeth.

### Laboratory Preparation of an Improved, Bi-Layer Clear Mini-Index

**Fig 3:**
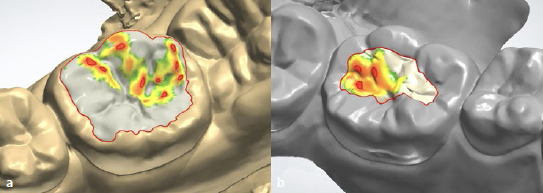
Digital wax-up of teeth 46 (a) and 36 (b). Red and yellow areas indicate the clearance of the opposing tooth.

**Fig 4:**
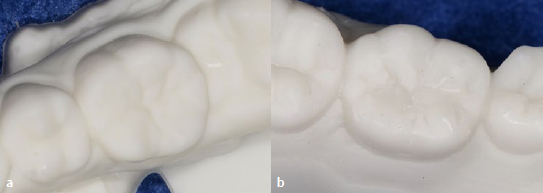
3D-printed casts of the digital wax-up of the teeth 46 (a) and 36 (b).

**Fig 5:**
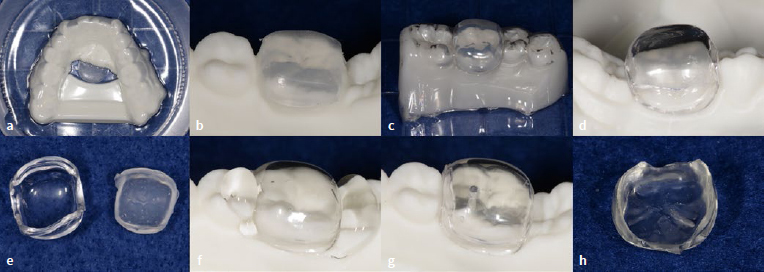
Fabrication of a bi-layer clear mini-index. a) A soft ethylene-vinyl acetate copolymer (EVA) thermoplastic sheet pressed onto the cast as a spacer for the clear silicone. b) Trimmed EVA sheet. c) The rigid polyethylene terephthalate glycol (PETG) thermoplastic sheet pressed over the EVA layer. d) PETG sheet was also trimmed. e) The outer PETG layer (left) was separated from the inner EVA layer (right). f) A clear silicone impression material placed in the PETG layer instead of the EVA layer and positioned on the 3D-printed cast. g) Prepared access hole in the occlusal surface. h) The completed mini-index.

In the laboratory, a digital articulator (Dental System, 3Shape A/X; Copenhagen, Denmark) was used to create the diagnostic digital wax-ups (Fig 3) and a mandibular cast was 3D-printed (Digital WAX 028D, DWS; Vincentza, Italy) (Fig 4). Bi-layer clear mini-indices consisting of an outer hard plastic shell and an inner elastic silicone material, were fabricated using the following process:

1. A soft ethylene-vinyl acetate copolymer (EVA) thermoplastic sheet (Erkoflex 3mm, Erkodent; Pfalzgrafenweiler, Germany) was used as a spacer for the clear silicone part of the mini-index. The sheet was pressed against the 3D-printed model using a pressure molding machine (Erkopress, Erkodent) (Fig 5a).
2. The trimmed EVA sheet was repositioned on the 3D-printed model (Fig 5b), and the rigid polyethylene terephthalate glycol (PETG) thermoplastic sheet (Erkodur, 2mm, Erkodent) of the outer layer was pressed onto it and trimmed (Figs 5c and 5d). The rigid sheet served as the outer part of the mini-index.
3. After separating the outer PETG layer from the inner EVA layer (Fig 5e), the space in the rigid sheet was filled with a polyvinyl siloxane (PVS) impression material (Exaclear, GC; Tokyo, Japan) and pressed onto the 3D-printed model (Fig 7f). For tooth 36, a less viscous silicone material (Exadenture, GC) was added to Exaclear to avoid air bubbles in pits and fissures.
4. Polymerization was performed in a dental polymerizer (Palmat Elite, Heraeus Kulzer; Hanau, Germany) at +0.2 MPa for 10 minutes to prevent air voids inside the PVS inner layer.
5. After polymerization, the mini-indices, consisting of a clear silicone interior and a rigid PETG exterior, were carefully removed from the 3D-printed model.
6. A single hole with a 1-mm diameter penetrating the index was made using a round diamond bur at low speed and cleaned using micro-brushes (Fig 5g).
7. The margin of the mini-index was trimmed using a diamond bur at low speed above the gingival level to avoid interference with the rubber-dam clamp during the intraoral restorative procedures, resulting in the completed mini-indices (Fig 5h).

### Restorative Procedure

**Fig 6:**
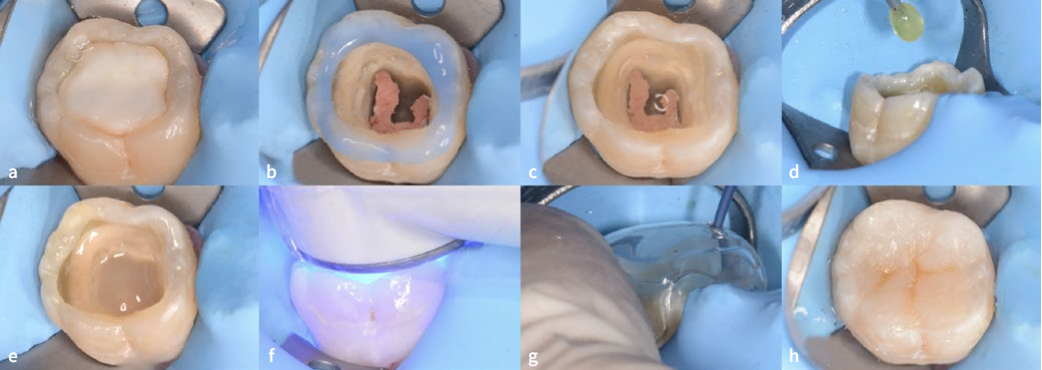
Restorative procedures of the endodontically-treated tooth 46. a) Preoperative occlusal cavity with a temporary glass-ionomer filling. b) Selective enamel etching after removal of the temporary restoration. c) Smear layer deproteinizing pretreatment using a 6% sodium hypochlorite solution, followed by a sulfinate-containing antioxidant. d) Two-step self-etch adhesive was applied. e) Application of the single-shade flowable composite after adhesive application. f) Light-curing of the lining flowable composite. g) Composite injection technique for the final layer placement using the bi-layer clear mini-index. h) Staining flowable composite was added into pits and fissures.

**Fig 7:**
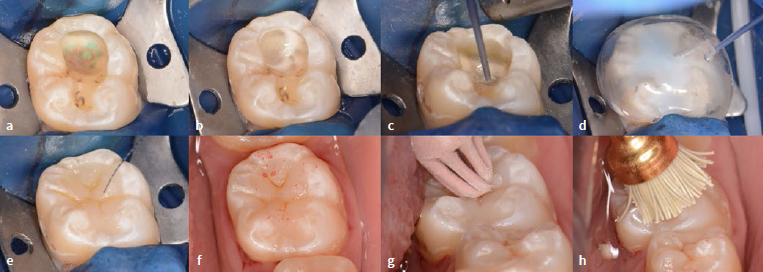
Restorative procedures of the stepwise excavated tooth 36. a) With the aid of a caries dye solution, selective caries removal to firm dentin was performed in the second visit. b) Smear layer deproteinizing pretreatment using a 6% sodium hypochlorite solution for 30 s. Note that the oxygen air bubbles are generated due to the decomposition of the sodium hypochlorite solution. c) After the application of an antioxidant and 2-step self-etch adhesive, a single-shade flowable composite was used. d) Inverse composite injection technique for the superficial layer. e) Staining flowable composite was added into pits and fissures to enhance the natural appearance. f) Occlusal contact evaluation using a registration paper. Note that the location of occlusal contacts corresponds to the digital simulation wax-up. g) Finishing using a diamond particle-containing rubber point. h) Polishing.

1. After the operative field was isolated using rubber-dam, the glass-ionomer cement (Fig 6a) was removed from tooth 46.
2. The occlusal enamel margins were selectively acid etched using 37% phosphoric acid gel (K etchant, Kuraray Noritake; Tokyo, Japan) for 15 s (Fig 6b), thoroughly rinsed with water and air dried.
3. A 6% sodium hypochlorite solution was applied to the dentin for 30 s to deproteinize the smear layer,^39,43^ followed by the application of an sulfinate-containing antioxidant (Clearfil DC Activator, Kuraray Noritake) for 10 s (Fig 6c).^17,36^
4. A two-step self-etching adhesive (Clearfil SE Bond 2, Kuraray Noritake) was applied according to the manufacturer’s instructions (Fig 6d).
5. A single-shade injectable RC (Clearfil SE Flow Universal, U shade, Kuraray Noritake) was incrementally placed in two 2-mm-thick layers (Fig 6e) and polymerized using an LED light-curing unit (Pencure 2000, J Morita; Saitama, Japan) for 20 s each (Fig 6f).
6. The prepared bi-layer mini-index was placed on the tooth, the same RC was injected through the access hole, and it was polymerized for 20 s (Fig 6g). After removing the mini-index, light curing was repeated for an additional 20 s.
7. A brown staining RC (Estelite Color, Tokuyama Dental; Tokyo, Japan) was placed and polymerized in the pits and fissures to mimic the natural appearance (Fig 6h).
8. A glycerin gel was placed on the occlusal surface, and light irradiation was performed again to promote a high polymerization degree of the staining composite.
9. Finishing and polishing were performed chairside in a relatively short time, using a 2-step polishing system (DIACOMP PLUS DCP-OFm, DCP-OFf, EVE Ernst Vetter; Keltern, Germany) and a polishing brush in which polishing particles are embedded (OptiShine, Kerr; Orange, CA, USA).
10. For tooth 36, the cavity was re-opened, and selective caries removal to firm dentin was performed (Fig 7a). The cavity was then restored using the above-mentioned technique (Fig 7b-h).

**Fig 8:**
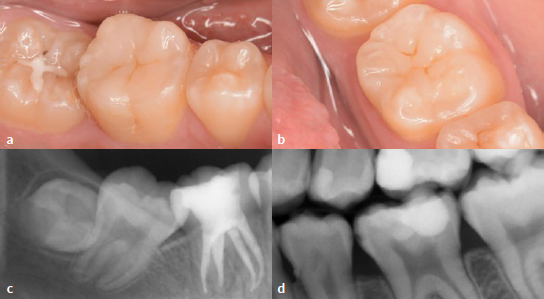
One-year follow-up. Intraoral views of teeth 46 (a) and 36 (b). (c) An apical radiograph of the restored tooth 46. (d) A bitewing showing the restoration of tooth 36.

Orthodontic treatment was performed after the restorative procedures. At a check-up after one year, the restorations presented marginal integrity without gaps and secondary caries. There were no cracks in the enamel, and no chipping of the RC was found. The surfaces were smooth, and no marginal discoloration was observed (Figs 8a and 8b). The patient reported no dentin hypersensitivity. The radiographic examination did not reveal any abnormalities, such as periapical radiolucency in tooth 46 or pulp alteration in tooth 36 (Figs 8c and 8d).

## Discussion

Direct composite restorations have proven effective for the restorations of small cavities, such as teeth with Black’s class I-V cavities and non-carious cervical lesions.^14^ However, free-hand restoration of anatomical morphology in large cavities or endodontically treated teeth can be challenging.^5,9,10,37^ Better outcomes can be achieved by planning and simulating the appropriate morphology before treatment and then accurately transferring it to the treated tooth. The composite injection technique, which uses a highly filled RC and a clear silicone index, can be beneficial from this clinical perspective. The highly filled flowable RCs, also known as injectable RCs, have been shown to possess mechanical strength, wear resistance, esthetics, and long-term durability similar to that of conventional RCs, with comparable clinical outcomes.^3,23,26,28^ Single-shade injectable RCs are now also available, allowing acceptable shade-matching even without laborious shade selection and layering.^27^

To use the inverse injection technique with a single-tooth rubber-dam isolation, the silicone index needs to be trimmed and downsized. Given the elasticity of the clear silicone, this could hinder the dimensional accuracy of the restoration. To address this issue, a bi-layer clear mini-index was developed, using a rigid PETG thermoplastic sheet as an outer shell and a clear silicone as the inner layer. This bi-layer index enabled an accurate transfer of the final anatomical forms from the digital wax-up, including pits and fissures, thus reducing the time necessary for articulation and finishing compared to the free-hand placement. On the other hand, the fabrication of the bi-layer index is more expensive and laborious, and the workflow is not fully digital at this point. Given that the treatment is performed in two visits, this technique is applicable in the second appointment of an endodontic treatment or step-wise excavation, as presented in this case report. However, further development and optimization would be necessary to make the technique applicable in other clinical situations or class-II cavities.

For the long-term success of direct RC restorations, a durable resin adhesive-dentin bond is essential. To enhance the durability of self-etch adhesives, the smear layer deproteinizing pretreatment (SLDP) using sodium hypochlorite or hypochlorous acid has recently been introduced.^22,39,43^ By applying a deproteinizing solution, the organic phase of the smear layer is removed, thus enhancing monomer infiltration and interaction with the underlying dentin. The deproteinizing pretreatment was followed by the application of a sulfinate-containing antioxidant, which counteracts the adverse effect of chloramine-derived radicals on the polymerization of adhesives and improves the resin adhesive-dentin bond.^17,36^ In-vitro studies also suggest that the SLDP pretreatment can improve the durability of the resin adhesive-dentin bond by preventing the formation of the hybridized smear layer.^43^ However, clinical evidence supporting these findings is not available to date.

## Conclusion

Large occlusal cavities after endodontic treatment and step-wise caries removal were functionally and esthetically restored with digitally guided RC restorations using the inverse injection technique. The improved bi-layer clear mini-indices fitted the treated teeth even with single-tooth rubber-dam isolation, and transferred the anatomical form perfectly, thanks to the elastic inner layer and rigid outer shell.

## Clinical relevance:

The digitally guided composite injection technique using a bi-layer clear mini-index offers a precise and efficient alternative to time-consuming free-hand placement, especially in large occlusal cavities. The clear mini-index can be used even with single-tooth rubber-dam isolation, but its fabrication requires an additional visit.
